# Memoir on the General Characters of Rachitis; Read before the Royal Academy of Sciences of Paris, July, 1837

**Published:** 1841-01

**Authors:** Julius Guerin


### THE WESTERN JOURNAL OF MEDICINE AND SURGERY.

JANUARY, 1841.

Art. I.—*Memoir on the General Characters of Rachitis; read before the Royal Academy of Sciences of Paris, July, 1837.* By Julius Guerin, M. D.
Translated from the French for the Western Journal, by Thomas W. Colescott, M. D., of Cincinnati.

### ADVERTISEMENT.

I read this memoir before the Academy of Sciences in the month of July, 1837. That period, already remote, is not as might be supposed, that of my observations on rachitis. These observations date from 1834 and 1835. They form part of a general work on the deformities of the osseous system, which I presented to the "Concours" for the great prize of surgery of the Academy of sciences in March, 1836. The report of the Academy at this concours proves it. The same conclusions in fact are found there,* that the reader will discover at the end of this memoir. To establish this circumstance is no less useful to science than to the author. To science, for is it not a guarantee of the truth of the resnlts to which I have attained, to be able after five years of daily and repeated verifications to maintain them as I first announced them? To the author it is not more indifferent to fix clearly the date of his researces. When a matter is new and cultivated by a small number of persons, the epoch gives itself little trouble to inquire to whom the priority of certain observations belongs. It is only when a very great number happen to apply themselves to the study of questions which at first had been made the object of study by a few, that the interest of knowing to whom belongs such or such a view, such or such an idea, begins to manifest itself; but, before that, there may be, and there often are, partial or total reproductions of the original works, that embarrass subsequently those who wish to write the history of science in an impartial manner. How often, indeed, does it not happen that the discoverer cannot be distinguished from the plagiarist! Then they are associated together in the same discovery, as is done every day in matters of judicial appreciation. Desiring to prevent, so far as my labors are concerned, this half-way justice, very convenient for every one except those whom it dispossesses, I have thought it right to insist upon the date of the results announced in this memoir: that authentic date is, I repeat, the month of March, 1836, since all the facts concerned were committed to a work deposited in the Academy of Sciences the close of March, as the printed report of the Commission of the Academy at that meeting shows.

There still exists among physicians great difference of opinion in regard to what should be understood by rachitis, as well as in respect to the general phenomena of this affection, the nature and frequency of the deformities characterizing it, and the different species of alterations which it produces in the osseous system. Owing to the etymology of the word, the name of rachitis is commonly given to the deformities of the vertebral column; yet these deformities are rarely the product of the disease to which they are attributed. A great many authors, too, give this name to every species of softening of the osseous textures occurring in adults: like the case of the woman Supiot, reported by Morand, of the woman with nails observed by Morand the younger, and many other examples of softening of the osseous system preserved in the history of the science. Lastly, it is not rare to find physicians who confound rachitis with scrofula, or nnite them as causes of the same effects. Yet, most of the deformities of the spine, the disease of Mad. Supiot, a great

*See the Report of the meeting for the great prize of Surgery, p. 22, article Rachitis, in the published proceedings of the Academy of Sciences for the year 1837.

number of other histories of supposed rachitis preserved in the books, and scrofulous affections whatever they may be, neither belong to rachitis, nor are dependant on it remotely or directly. This uncertainty and obscurity have proved to me that few precise notions are entertained in regard to the malady to which the term rachitis, as established by Glisson, Mayow, and J. L. Petit, ought to be restricted. With the view of supplying this deficiency, I have devoted myself to multiplied researches upon the external characters of this affection, its mode of invasion and development with respect to the age, sex, and the parts of the osseous system that it successively occupies; the general phenomena which precede and accompany its evolution; the alterations of form and texture which it produces in the osseous tissue; the circumstances under which it is developed, and its presumed cause; in fine, I have sought to give precision to the diagnosis of rachitis in reference to the affections with which it has been confounded. I have examined separately each of these points as so many terms indispensable to the determination of the malady, and I am convinced that most of the descriptions hitherto presented for one and the same disease, have joined in a confused manner characters proper to several diseases, and neglected precisely those that belong specially to rachitis. It is the result of these inquiries, pursued attentively for several years amongst a great number of subjects differing in age, sex, and conditions, that constitutes the object of this memoir.

### Sec. I.—*General Forms and Characters of Rachitis upon the Skeleton and the Living Body.*

In order to have at once a general idea of rachitis, and embrace at a single glance the assemblage of external forms and characters which this affection assumes in its complete manifestation, it is sufficient to examine in turn the skeleton of a person who has experienced it in a marked degree, and a living individual who presents it in its most advanced period.

*In the skeleton*, the whole osseous frame-work, from the bones of the feet to those of the cranium, is seen to have undergone numerous deformities, and changes of proportion and direction: the bones of the members are regularly curved like an arc, and more or less twisted on their axis, particularly the tibia and fibula, then the femur, and in a less degree the bones of the superior limbs: the extremities of these bones are swollen, rounded, enlarged and deformed: the articulations of the feet and hands offer anormal directions and relations: the apophyses and points of muscular attachment are salient: the ribs are rounded as well as swollen, and present at their sternal extremity a curvature whose concavity looks forwards and outwards: the thorax is thus flattened transversely, and depressed in the form of a trough level with the articulations of the ribs with the sternal cartilages.

The vertebral column deviates laterally or posteriorly, and presents a succession of alternate curvatures. The surfaces of the vertebræ are rounded, as it were distended and swollen, and present in several points depressions which have approximated the articular surfaces of their bodies. The vertebral appendices participate in the same appearances, and are more or less depressed as if crushed in the direction of vertical pressure and muscular action. The pelvis has lost its symmetrical conformation; the sacrum is sometimes vertical, at other times more crooked than in the normal state, and it offers an unsual thickness. The cocygeal bones are more or less approximated and forced upwards and inwards; they are thicker in every direction, and participate in the enlargement affecting all the spongy bones. The same is true of the scapula and sternum: they offer an anormal configuration, are shortened, swollen, and thickened and curved on the flat side. The head is generally voluminous, the cranium being thickened, especially upon the sides. The bones of the face, owing to the increased development, present more marked saliences, and sharper and more prominent angles. The relations of proportion of every part of the osseous system are generally perverted: the skeleton, as a whole, like each of the bones composing it, offers a true reduction in length and in extent, and gives evidence of an arrest of development. Thus, viewed upon the skeleton, rachitis evidently consists in a general affection of the bony system, occupying all its divisions, diffused through its smallest appendages, and discovering itself without by a general swelling of the epiphyses and spongy bones, and by the curving of the long bones and vertebral column; the whole accompanied with a character of general alteration of the surfaces that gives to the osseous tissue an appearance of thickening as if all its component parts had been swelled, distended and softened by the presence of a foreign substance; and an appearance of general deformity, as if the weight of the superior parts, and muscular contraction, had impressed their influence incessantly upon every part according to the direction and proportion of their action.

*Upon the living body*, the prominences and deformities of the skeleton can be distinguished more or less easily. The face is characteristic: the prominences of the cheek bones very marked, the features wan. The subject has the physiognomy belonging to a more advanced age; the eyes are very open, lively and brilliant, the wings of the nose dilated, the mouth wide and gaping. The head is thrust down as it were between the shoulders. The chest is narrow, contracted, flattened or depressed laterally. The clavicles, following the approximation of the shoulders, increase their curvature. The trunk and limbs are thrown into a heap; the articular extremities, the tarsus and the carpus particularly, are swollen; all these parts are as it were retained in one another, and as if arrested in their development, whence our ancestors said correctly that rachitic children were *knotty* (noués.) The whole being is shrunken and stunted. The skin is generally dull and earthy; that of the face is sometimes pale and slightly violaceous; sometimes, on the contrary, it is colored and somewhat blotched with red; this last peculiarity is observed chiefly among the children of the wealthier classes, which present the disease in a feeble degree only, such as the deviation of the knees, or the incurvation of a leg, without any very marked general reaction. In the rest of the body the skin presents a pale, dull color, offers numerous folds in the concavities of the curvatures,* and at the swollen articulations; the pilous system is developed principally along the vertebral column. The muscular system is thin, the muscles are short and as if struck with an arrest of development. The motions are constrained and difficult, the walk painful, reeling, and often completely impossible. The subjects avoid standing on the legs. Respiration is short and laborious; is hardly thoracic, but diaphragmatic or abdominal. The abdomen is large, tense, but not painful; the skin warm and moist. The subject perspires a great deal, especially about the abdomen and head, and chiefly during sleep; the sweats are remarkably acid. The appetite is feeble, the thirst pretty great. Di-

*Whenever this term occurrs hereafter, I shall use the word *lesser curvature*.

gestion is difficult; there is nearly always diarrhœa, or if it disappear, it alternates with constipation and returns with the greatest facility. The stools are serous, the urine abundant and but little colored. The pulse is small and frequent, almost febrile. The impulse of the heart is feeble. Intelligence is ordinarily active. The sensibility is exquisite and the subjects cry on the least touch, although there is no point particularly painful. The bones of the limbs are bent or incompletely fractured with the greatest ease; there are some cases even in which muscular contractions alone saffice to bend them.

Such is rachitis seen in the assemblage of its external forms upon the skeleton, and of its general characters upon the living body: presented as it has just been, this description is an abstract of the beginning, mode of invasion, course, periods, and degrees of the malady; it is composed only of the prominent and perfect features, without considering their mode of development, and above all without determining the immediate characters of the osseous tissue. I shall now examine those different combinations as they are found in nature, and as I have verified them in the subjects submitted to my observation.

Sec. II.—*Mode of Invasion and Development of Rachitis; Influences relative to the age, sex, and the parts that it affects.*

Rachitis properly speaking is a disease of infancy; it is rarely seen in the fœtus, most frequently towards the age of from eighteen to twenty months, very seldom after the age of puberty. In three hundred and forty-six cases examined by me in reference to this point, the invasion occurred as follows:

| | | |
|---|---|---|
| Before birth, - - - - - - | 3 | cases. |
| In the course of the 1st. year, - - - | 98 | " |
| " " " 2d. " - - - | 176 | " |
| " " " 3d. " - - - | 35 | " |
| " " " 4th. " - - - | 19 | " |
| " " " 5th. " - - - | 10 | " |
| From the 6th to the 12th. " - - - | 5 | " |
| | 346 | " |

It attacks both sexes with nearly equal frequency. Of these three hundred and forty-six cases, there were

| | |
|---|---|
| Males, - - - - - - - - | 148 |
| Females, - - - - - - - | 198 |
| Total, - - - - - - - | 346 |

The knowledge of these two orders of facts relative to the influence of rachitis in respect to *age* and *sex* offers already two valuable characters for showing that certain diseases, and many deformities of the vertebral column that have been attributed to rachitis, are the product of other causes: so that every species of softening of the bones in adults, and all deformities which supervence almost exclusively in young girls towards the age of puberty, are not caused by this malady. The truth of these conclusions will appear more clearly in proportion as I make known the true characters of the affection.

*Mode of Invasion and of Development; Periods.*

We must not suppose that rachitis commences at the moment only of the deformity of the bones. This manifestation of the disease belongs to an order of secondary facts. Before passing into deformity of the osseous system, it is announced by some more or less perceptible general phenomena, that are wanting in those cases only in which the affection is slightly marked. These phenomena are gastro-intestinal derangements, diarrhœa, swelling of the abdomen, nocturnal sweats, a febrile disturbance, a feeling of weakness, and a marked sensibility of the whole osseous system. The individual becomes melancholy, and morose; his features alter and assume a withered look; the muscular system loses its consistence, and the whole being seems to be affected silently by a general morbid cause which has as yet no other external manifestation than these general phenomena. Towards the end of this first period, which I call that of *incubation*, or *effusion*, as will be seen hereafter, the articular extremities swell; there is yet no curvature, and it may even happen that there may be none in the whole course of the malady. I have observed several examples of well marked general rachitis in which the long bones, and vertebral column preserved a remarkable rectitude. The swelling of the epiphyses and the shortening of the long bones were the sole physical characters of the affection which ran through all its periods without producing incurvation of the bones of the members or of the vertebral column. Nevertheless it is far from being thus in the generality of cases. Almost always the deformity of the skeleton succeeds immediately the period of incubation.

The duration of this period is from two to six months. I have seen three children in whom it was almost imperceptible. The bones had become crooked without the health of the subjects appearing to have previously suffered. These instances are rare. In the large majority of cases the characteristic phenomena of this period are among the most manifest. Yet we must beware and not mistake the effect for the cause: most rickety children have been badly nourished, badly lodged, badly aired, and these anti-hygienic conditions have developed in them a state of languor, disorder, and susceptibility in the digestive functions. The true characters of the period of rachitic incubation are the nocturnal sweats of the belly and head, doughiness (emhâtement) and swelling of the belly, diarrhœa without griping, a moist heat of the skin, a constant and uniform febrile movement, and lastly a great sensibility of the osseous system, and an aversion on the part of the patients to the erect position.

The period of deformity is announced ordinarily by the swelling of the ankles, the knees, and the wrists; these articulations acquire nearly double their ordinary volume; they are knotty-like, and between their surfaces of junction, for instance between the wrist and forearm, between the leg and the foot, the skin forms a deep fold which betrays the excess of development of the articular extremities covered by it, and their want of immediate conformity. Very soon the bones of the legs bend, the knees deviate, and the thigh-bones, hips, chest, vertebral column, and the other portions of the skeleton partake of the general alteration of form. Whilst the local symptoms become more and more marked, the general symptoms augment in a sensible manner: the belly continues large; a great deal of gas accumulates there, and sometimes a quantity of liquid appreciable on percussion. The diarrhœa persists and frequently augments; the urine and sweats become more and more copious; the respiration is frequent, laborious, and purely abdominal; the febrile movement is characterized by a sensible heat of the skin and a uniform acceleration of the pulse, such as are observed in the continued fevers of con sumption. The sensibility of the osseous system increases; the patients cry on the slightest compression of their limbs; all the functions languish. The constitution becomes enfeebled, the color fades, the muscular system becomes impoverished; standing and walking are impossible, and are suspended during the greater part of this period. At the same time that the deformity of the osseous system is augmenting, the disease arrives at its height of intensity. This period lasts from one to three years, and it is during this space of time that we see the distortion of the different parts of the skeleton successively take place. This distortion is not always general, or at least it is not perceptible in all the dependencies of the osseous tissue. For instance, the disease is sometimes limited to the swelling of the epiphyses, particularly to the swelling of the articulations of the knee, the malleoli and the wrist; to the deviation of the knee; to incurvation of one or both legs; to swelling of the epiphyses, with flattening of the chest or curvature of the spinal column: I have observed all these combinations.

*Proportion and order of succession of the Deformities.*

It was a subject of curiosity to know in what proportions and in what relations these various deformities appear, and in what order they develope themselves, or succeed each other. Here is the result of my observations in this respect:

In four hundred and ninety-six cases of rachitis there were

| | | |
|---|---|---|
| With general symptoms and swelling of the articulations, without curvature, - - | 11 | cases |
| With deformity of the inferior limbs and swelling more or less marked of the wrists, - | 485 | " |
| Total, - - - - - - - | 496 | " |

Among this number, deformities occurred simultaneously

| | | |
|---|---|---|
| In the continuity of the superior members - | 14 | times |
| " " of the vertebral column, - | 48 | " |
| " " of the thorax, - - - | 59 | " |
| Apparent development of the cranium, - - | 17 | " |

Whence it follows that rachitis seldom adheres to the development of the epiphyses without a curvature of the diaphyses; that the superior members show by the swelling of the wrists their participation in the general affection, but that they are rather infrequently distorted; that the inferior members are so nearly always, and that the deviations of the spine are to the deformities of the inferior limbs in respect to frequency about as one to ten. The proportion of the deformities of the chest is a little higher, and the development of the cranium a little less frequent.

As to the order of succession of the deformities it appears to be as follows: swelling of the epiphyses of the lower limbs, deviations of the knees, curvatures of the tibia and fibula, curvatures of the femur; next the enlargement of the wrists, and simultaneously or consecutively the distortion of the pelvis; then swelling and distortion of the ribs, scapulæ, clavicles, and the deviation of the spine; the development of the cranium and swelling of the facial bones take place last. This order of succession is incontrovertible. In forty-two cases that I have observed and attended for several years in Paris, the rachitis manifested itself forty-one times by the swelling of the articular extremities and curvature of the inferior limbs: in one only, the affection seemed to commence by the deviation of the spine; in none by the distortion of the thorax.

The interval which intervenes between the development of the deformity of the spine, or of the thorax, and that of the inferior members, varies from one to three years. In by far the greater number of cases, nevertheless, the spinal deviation has discovered itself about one year after that of the limbs. It is an unquestionable law, however, and one that scarcely admits of an exception, that rachitis proceeds in the distortion of the skeleton from below upwards, and that the deformity of the spine is the last to manifest itself. These observations upon the order of development of rachitic deformities, are further confirmed by the following, namely; that the degree of these successive deformities is ordinarily in conformity with their order of manifestation. Thus the bones of the legs are generally more deformed than the thigh-bones; the latter a littler more than those of the pelvis; these in their turn more than those of the superior members and the thorax; then come almost on the same line, the distortion of the vertebral column, and the degree of enlargement of the cranial bones. The deformity the most important to describe in its relations of succession and of degree was that of the pelvic bones: now I have remarked that the distortion of these bones was always accompanied by that of the bones of the inferior extremities, and that the degree of deformity of the latter, compared with that of the bones of the superior extremities, expressed pretty well the degree of deformity of the pelvic bones; whence it follows,

1. That all rachitic deformity of one portion of the skeleton implies that of those placed beneath it: for instance, that of the vertebral column implies that of the pelvis; the latter, that of the thigh-bones, that of the tibiæ and fibulæ, and the first implies the last;

2. That the degree of each deformity is in relation with the order of succession to which it is subjected, that is to say, the degree of the deformities diminishes from below upwards;

3. That every isolated deformity of one of the superior portions of the skeleton, of the vertebral column, for example, without deformity of the parts situated beneath, is not due to rachitis.

When patients have arrived at the height of the period of *deformity*, a third order of phenomena manifests itself, constituting a third period of rachitis, which I call that of *resolution* or *consolidation*. It is announced by the progressive diminution of the symptoms belonging to the preceding period. Thus the pulse loses its frequency, the diarrhœa diminishes and even ceases altogether. The abdomen gradually loses its hardness and its fulness: the urine is discharged less frequently, and of a higher color; the sweats become much less, and it is principally by this symptom we recognize that the disease is in its period of resolution or of consolidation. The skin is less hot and less humid; it resumes its healthy color. The face is no longer contracted, and the complexion is animated. In a word all the functions successively resume their activity; the digestion, the respiration, the circulation are restored and made regular; the muscular forces return, and the patient, more confident in himself, and above all freed from that sensibility and feebleness of the limbs, resumes the exercises of standing and walking, often after having suspended them for a long time.

On the part of the osseous system, this period of the disease offers some phenomena which it is no less important to note. The epiphyses lessen sensibly in volume; we may sometimes verify a diminution of from one to two inches in the space of three or four months. The complete resolution of the epiphyses may thus take place by the efforts of nature alone. It is not so with the deformities of the skeleton; they often diminish of themselves, but rarely disappear completely without the aid of mechanical means; I have observed, notwithstanding, several examples of spontaneous correction of curvatures of the limbs under the influence of a general treatment; but the most frequent occurrence, especially when the curvatures have been carried to a certain degree, is the persistance of the deformity, and the consolidation of the bones in this anormal disposition. It is remarkable that after the period of resolution the deformities of the limbs no longer augment, but then, too, they no longer diminish of themselves. The bones that, in the preceding period, were soft, and flexible by the least efforts of the hand, have become hard, solid, and completely inflexible, whatever effort one may make. Having arrived at the end of this period of the malady, the subjects are healed, or approach a complete cure, but they retain their deformities.

The termination of rachitis does not always take place by complete resolution. Some patients in whom the affection has been carried to a considerable degree, which is betrayed by the universality and exaggeration of the deformities of the skeleton; these patients, I say, preserve during part of their life, and even through the whole of it, traces of the profound disorder which has affected their skeleton. In them the rachitis has passed into the *chronic state*. The signs proper to this phase of the malady no longer offer any analogy with those of the three periods of the acute affection; there is neither fever, nor sensible alteration of any of the principal functions. The chronic rachitis manifests itself by two constant and pretty decided symptoms, a fatty (graisseuse,) frail constitution, made evident exteriorly by a pale and leaden complexion, and a great fragility of the bones. This fragility is such that the compression of certain bones between the fingers is sufficient to break the external table, which is not thicker or more consistent than an egg-shell.

Such are the external phenomena characterizing the three periods of rachitis:—its period of incubation, of deformity, and of resolution or consolidation. The knowledge of these phenomena leads already to some general results, several of which are entirely new, and others have not been well settled: these results may be summed up as follows:

1. Rachitis is exclusively a disease of infancy;

2. It offers a period of incubation characterized by some general symptoms which do not as yet belong to the affection of the osseous tissue;

3. Considered in its effects upon the bony textures it is a general malady of the whole skeleton, which manifests itself however in different degrees and under different forms in each portion of the skeleton;

4. That rachitic deformities of the skeleton proceed successively from below upwards, from the bones of the leg to the thigh-bones, from the latter to those of the pelvis, from the pelvic bones to those of the superior limbs and of the thorax, and lastly the spinal column and cranium;

5. The degree of the deformities is in relation with their order of development: the deformity of one portion of the skeleton implies always that of the other parts situated beneath it;

6. Rachitis, considered in the development and the series of its phenomena, offers distinct periods during which the disease changes more or less in aspect and character.

Let us pass now to the history of another order of facts, to the examination of the immediate characters of the osseous tissue; this examination will only serve to establish more firmly the foregoing conclusions.

### Sec. III.—*Immediate Characters of the Osseous Tissue.*

The immediate influence of rachitis upon the osseous system is revealed by four different orders of facts: by the *deformity of the bones;* their *reduction in dimensions;* the intimate *alterations* of their tissue; aad lastly, by the *disorder* and *delay* it occasions in the progress of ossification. The facts relative to the deformities of the skeleton have been noticed at all times; I have nothing to add for the moment to the general indications that I have heretofore given; I shall recur to it in detail when giving the particular history of the different orders of deformities produced by rachitis. I pass at once to the three orders of facts: the *reduction in dimension of rachitic bones*, the *alterations of their tissue*, and the *influence of rachitis upon the progress of ossification.*

### A.—*Reduction in the Dimensions of the Bones.*

I have collected a certain number of skeletons, offering evident traces of rachitis, from the Dupuytren Museum, the Museum of Clamart, and of La Maternité, which I have added to several of my own. I have measured with the greatest care the relative dimensions of the bones of the superior and inferior members, in comparison with their length in the healthy state; the comparative dimensions of other bones of the skeleton, of the pelvis, vertebral column, scapula, clavicles and sternum; from my researches committed to the table placed at the end of this memoir there results:

1. That most of the bones of the rachitic, compared with those of the normal skeleton, are affected with an arrest of development in regard to their different dimensions.

2. That this reduction, independently of what results from the deformity of the bone, may amount to one-half the ordinary length of the bone.

3. That this reduction is in nearly a constant relation with the order followed by the disease in its development; in other words, that the reduction of the bone is generally so much the greater as the portion of the skeleton is more inferior, and that it diminishes gradually from below upwards; from the bones of the legs to the thigh-bones, from these to the pelvis, from the pelvis to the superior members and the spinal column, etc., so that the normal relation established between the length of the upper and lower limbs diminishes in favor of the former.

4. That the reduction of the inferior members by comparison with that of the superior members tends to establish that rachitis has the effect of perpetuating, by a more sensible arrest of development of the inferior members, the relations that existed between the superior and inferior members, at the time when the affection seized them.

These various results flow rigorously from the numerical relations that I have consigned in the three tables placed at the end of this memoir. It is there seen, in fact, that in each of the cases which have been analyzed the bones affected with rachitis were never so long as the healthy bones, and that their mean length especially was sensibly less than the normal mean length. (See Table No. 1.)

I have taken for a term of comparison the skeletons of rachitic females exclusively, in order to have more rigorous relations, and such as conduct to more direct practical applications in what concerns the measurements of the pelvis.

Thus, Table No. 1. in three rachitic female skeletons the mean length of the bones was as follows:

| | Rachitic Bones. | | Normal Bones. | | Difference. | |
|---|---|---|---|---|---|---|
| 1. Peroneus, - - - | 9*in.* | 5*l.* | 13*in.* | 2*l.* | 3*in.* | 9*l.* |
| 2. Tibia, - - - | 10 | 1 | 12 | 6 | 3 | 5 |
| 3. Femur, - - - | 10 | 11 | 14 | 0 | 3 | 1 |
| 4. Radius, - - - | 6 | 8 | 8 | 4 | 1 | 8 |
| 5. Cubitus, - - - | 7 | 6 | 9 | 3 | 1 | 9 |
| 6. Humerus, - - - | 8 | 11 | 10 | 6 | 1 | 7 |
| 7. Clavicles, - - - | 5 | 2 | 5 | 8 | 0 | 6 |
| 8. Sternum, - - - | 5 | 0 | 5 | 5 | 0 | 5 |
| 9. Column, - - - | 20 | 11 | 22 | 0 | 1 | 1 |
| 10. Three diam'rs of the Pelvis, | 11 | 9 | 13 | 6 | 1 | 9 |

This comparison of the different parts of the skeleton shows not only that the reduction of rachitic bones is affected from below upwards, but that it occurs in each of them compared with the corresponding ones of the normal skeleton, according to a series of pretty regularly decreasing numbers, as may be seen by the following indication of mean lengths extracted from my first table:

| | | | | | |
|---|---|---|---|---|---|
| Peroneus, | 28*in.* in 100 | Radius, | 20*in.* in 100 | Clavicles, | 9*in* *in* 100 |
| Tibia, | 25 " | Cubitis, | 19 " | Sternum, | 8 " |
| Femur, | 22 " | Humerus, | 15 " | Column, | 5 " |
| The three diameters of the Pelvis, - - - | | | | | 17 " |

Thus 28, 25, 22, 20, 19, 15, 9, 8, 5, is the formula of the decreasing reductions of rachitic bones from below upwards.

Whence it follows:

1. That the reduction of rachitic bones is a general fact which is effected according to the same law as their deformity, successively from below upwards, and gradually from above downwards.

2. That the dimensions of a rachitic bone being known, the dimensions of the other parts of the skeleton may be approximately determined.

3. That the reduction of the three diameters of the pelvis in rachitic females follows that of the parts composing it, and that the degree of this reduction is intermediate to the degree of reduction in the femur or the humerus.

As to the relation existing between the length of the upper and lower extremities affected with rachitis, considered as perpetuating the relation existing at the time the affection invades the osseous system, it is a fact as unquestionable as the preceding. I have demonstrated that the mean of the most frequent invasions of rachitis corresponds to the age of from eighteen months to two years; now, according to a calculation that I have made of the comparative length of the superior and inferior members at this age, I find, as any one will be convinced by casting his eyes over Table No. 2, that in the healthy infant of two years, the length of the superior members is to that of the inferior as 1 to 1. 34. This relation in the rachitic adult is 1. 35, whilst in the healthy adult it is 1. 46. It will be seen, therefore, that in this circumstance, rachitis, acting as a cause of the arrest of development, gives rise to effects analogous to those produced by the causes which impede the evolution of organs in the fœtus, that is to say, by perpetuating the phase of development corresponding to the time of arrest. I need not lay down here the physiological reason which accounts for the relative inferiority of the lower extremities of children of tender age; it is a fact belonging to science that needs only be cited in order to be admitted with the authority of a demonstration.

Thus far I have considered the reduction of rachitic bones only in its most perfect manifestation upon the skeleton of adults. Now this fact, observed at so remote a period of the malady, might be considered as a consecutive effect of its influence, and not as one of its direct characters, the more so as some authors who have written upon rachitis, deny the existence in this malady of an arrest of development of the bones. To solve this difficulty and give to my observation all the precision of which it was susceptible, I have submitted the various parts of the skeleton of several rachitic children to rigorous measurements repeated at an interval; for instance, I have taken the dimensions of the bones of the leg, the thigh, the forearm, the arm, the vertebral column and of the entire waist, repeating these measurements at an interval of several months, and have obtained the knowledge of these two facts, to wit:

1. That children affected with rachitis from about the age of one year, are smaller at the waist, and have every part of the skeleton generally shorter, than healthy children of the same age: (Table No. 3.)

2. That during the first two periods of the disease, the growth of the bones in length is completely arrested in a certain number of cases; in some others it continues, but, generally, in a manner perceptibly slower, and in particular slower in the inferior extremities than in the superior. In some cases, children affected with rachitis in a marked degree, have ceased to grow for eight or ten months; others have continued to grow, but not in a very sensible manner. Lastly, in comparing the degree of increase of the inferior with that of the superior members, I have observed some cases in which the superior alone had continued to be developed, whilst the inferior had remained stationary, but I never observed the contrary. This fact of the want of growth, or simply of a tardiness in the growth of the bones in length, has appeared to me subordinate to the influence of the degree of the malady; with a highly marked rachitis, the growth was completely suspended; with a less degree, it was only diminished. The relation which this fact sustains to the termination of the rachitis, confirms again its subordination to the direct influence of this malady. For instance, as soon as the period of consolidation or of resolution has commenced, the growth of the bones in length is renewed, but nearly always with less activity than in individuals who have never had rachitis; and this tardiness of increase continues until the cessation of the development of the skeleton, so that the sum of the reduction which the long bones present in rachitic adults is composed of two added results: of the reduction proceeding from a true arrest, or simply from a diminution of increase during the malady, and from a diminution of increase subsequent to the malady. I think, moreover, that to the influence of the degree of the affection, considered as the immediate cause of the more marked arrest of development, it is proper to add the influence of the age at which the disease made its invasion; for the rachitic adults who have remained the smallest are those who had been attacked with the disease at a more advanced age, at the fifth, sixth, or seventh year.

I have imagined I could see, moreover, in these rachitic individuals, that the bones had less tendency to become crooked, and that the disease betrayed itself chiefly by the swelling of the epiphyses and the shortening of the long bones.

Such are my observations relative to the reduction of rachitic bones. They may be recapitulated thus:

1. Most of the bones of the rachitic skeleton are always relatively less developed in length and in breadth than those of the normal skeleton.

2. This reduction, independently of that which results from the deformities, may be carried to more than half of the normal dimensions.

3. The reduction of the rachitic bones is effected according to the same law with their deformities, successively from below upwards, and gradually from above downwards.

4. The decreasing series of the rachitic reductions from below upwards is represented by a regular series of numbers which permit us, the dimension of one bone being known, to determine the approximation by that of other parts of the skeleton.

5. The reduction of the three diameters of the great pelvis follows that of the dimensions of its component parts; the degree of this reduction may be calculated by means of the reductions of the femur and humerus.

6. The greatest reduction of the inferior compared with that of the superior members establishes between these parts relations of lengths which repeat and perpetuate exactly those of the age at which the malady was developed.

7. The reduction of the bones considered in rachitic adults is the result composed of the arrest of development of the osseous system under the direct influence of the disease, and of the consecutive slowness of its increase subsequent to the disease.

8. The reduction of rachitic bones is a general, and as constant a character of the malady as their deformity.

B.—*Alterations of the Texture of Rachitic Bones.*

The question of the alterations of texture of rachitic bones, has frequently, and for a long time, occupied the attention of anatomists. Among all the authors who have published their observations on this important point, there are not two who agree in the results arrived at. This diversity of opinion has seemed to me a proof of the inaccuracy of most of them, and I have explained it by a cause which nearly always accounts for the dissentions and contrariety of opinion that prevail amongst men who observe and study patho-iogical facts. This cause is too clearly demonstrated, in what concerns rachitis particularly, for me not to indicate it here, the more so as it will have the effect of making us admit definitively whatever is true in the observations of those who have preceded me.

All the authors who have accounted for the alterations of texture of rachitic bones, have considered rachitis as an affection of the same kind at all epochs of its development, allowance being made for its periods, degrees, and acute or chronic form. When opening a rachitic bone the alteration which they supposed they saw was, in their view, the single characteristic of the disease, and if this fact was in opposition to those formerly indicated, the latter were declared figurative, hypothetical, and regarded as never having occurred. And yet the alterations of textures of the osseous tissue in rachitic individuals have their phases, their commencement, degrees, periods, and termination. Bound to the disease, of which they are the partial manifestation only, they follow the course of its development, commencing, increasing, diminishing, disappearing, or perpetuating themselves with certain appearances that vary like the other characters and symptoms of the affection which produces them. It is difficult to understand, indeed, how this error could be committed, and yet it has been nearly always in the observation of all diseases, at least in the origin of their study. What follows will prove that there was still this confusion of facts in respect to rachitis.

After having opened and examined a large number of rachitic bones, I have arrived at the conclusion that the alterations of texture which they present are positively different according as they belong to the period of incubation, of deformity, or of resolution, and according as the disease is completely resolved or has passed into the chronic state. I shall point out the different alterations proper to each of these phases of the malady which I shall arrange in two categories, the first comprising every period and degree of *recent* or *acute* rachitis, and the second every period and degree of *ancient* or *chronic* rachitis.

### a.—*Texture of the Bones in Recent or Acute Rachitis.*

1. Alterations of texture proper to the period of rachitic incubation.—It is rather difficult to procure bones belonging to this period of the malady; first, because the deformity of the skeleton does not yet exist, at least in any very sensible manner, and, secondly, because rachitis at this period does not seem capable of producing death. Meanwhile it is not rare to see children, who are already enfeebled by a debilitating affection like rachitis, fall victims to other acute intercurrent maladies, such as pneumonia and acute hydrocephalus. The whole difficulty in these cases is to distinguish the symptoms which are proper to rachitis, and which announce it, in the midst of those proper to the intercurrent affection. These symptoms, among other phenomena less marked and less important, are a diarrhœa of long standing, copious nocturnal sweats, an exalted sensibility of the limbs, the repugnance of the children to, and the difficulty they experience in sustaining themselves on the legs; and lastly, a slight swelling of the articulations. I will add, moreover, that it is not infrequent to find in patients whose lower limbs are affected with the characteristic deformities of the second period, the bones of the superior members, the humerus particularly, with the alterations proper to the period of incubation. Here is what these alterations consist in:

When a rachitic bone of the first period is divided into two longitudinal halves, a remarkable quantity of sanguinolent matter is found effused in the medullary canal and at the level of the epiphyses, distending the areolæ of the spongy tissue. This matter is not of a lively red like ordinary blood, but a little deeper. By compressing the cells of the epiphysary extremities the effused liquid flows out in abundance. A more careful examination shows that a certain quantity of this matter exists between the periosteum and the external lamina of the diaphysis, and between the medullary membrane and its internal osseous lamina. A third more important fact is remarked, the presence of the same liquid between several lamellæ of the compact tissue. This last circumstance can be perceived with the naked eye; when examining the surface of the divided bone, a series of more or less continuous, reddish, parallel lines is distinguished, which are due to the overflow of the sanguinolent liquid. This fact becomes more and more appreciable when the periosteum is first removed, then the successive layers of the compact tissue, which are easily enough detached. By this means, one can expose an entire layer of the liquid effused between two lamellæ, and study its characters. At first it is of an aqueous consistence, and may be removed by washing with water, as well as by desication; it seems to be the same between the periosteum and first osseous layer, between the medullary membrane and the internal layer. At a more advanced stage the same substance gives place to a deposit of a gelatiniform matter, which organizes itself after the manner of false membranes, that is to say, myriads of small vessels are discerned in it, intercrossing and forming an inextricable net-work. These vessels may be distinguished when magnified twenty-five diameters only; having reached this degree of organization, the effused matter becomes adherent to the surfaces with which it is found in contact, and it is no longer possible to isolate it by means of ablution. The periosteum is manifestly injected and a little thickened; injection pushed into its capillaries developes arborizations there, very numerous, and more sensible than in the healthy bone. The nutritive vessels of the bone are remarkably developed and the effused liquid is particularly abundant at the level of their principal divisions. The medullary membrane is sometimes thickened, but less frequently and less sensibly than the periosteum. The compact tissue of the bones is not yet sensibly softened, still it no longer breaks as easily and as completely as in the healthy state. The concentric lamellæ, already removed from one another by the presence of the effused liquid, can be separated with sufficient ease: dried, they allow a multitude of small openings to be seen, the more marked and more appreciable as the osseous layers approach nearer the periosteum; the areolæ of the spongy tissue are dilated, and a certain number are sometimes found united, as if their partitions had been destroyed by the presence of an excessive quantity of effused matter.

The alterations I have just described belong to the long bones. The tibia, the fibula, the femur, and the bones of the superior members generally exhibit them. Those which are observed in the spongy and short bones are analogous: for instance, in the first stage of the period of incubation, the bones of the pelvis, the scapulæ, and even sometimes the cranial bones, are the seat of a remarkable sanguineous effusion, which distends their cells and enlarges them considerably. It is the same with the bones of the tarsus, carpus, and with the vertebral bodies. Nevertheless the effusion seems less abundant in the latter than in the long bones and their epiphyses.

Such are the immediate characters of the osseous tissue in the first period of rachitis. These characters may be included in one general fact, namely; a general effusion of blood, made less viscous and less consistent, in all the interstices of the osseous system, which is as it were infiltrated.

### 2.—*Textural Alterations of the Second Period of Rachitis.*

When the long bones affected with rachitis at the second period, that is to say, offering pretty marked, but recent deformities, are opened, here is what is encountered: towards the epiphyses at their union with the diaphysis, a consistent and elastic spongy substance having very fine, close meshes is perceived, which does not result from a transformation of the ordinary spongy tissue, but is a pathological addition of recent formation, placed either at the exterior of the normal spongy tissue, or in the cells themselves of this tissue, in greater quantity, however, at the point of junction of the epiphysis with the diaphysis. This newly formed tissue is not found only at the extremity of the long bones; its existence is as general as that of the cause from which it takes its origin. I have remarked that towards the end of the first period, the matter effused in all the osseous tissue, particularly that furnished by the periosteum and that deposited between the concentric laminæ of the long bones, presents some traces of organization apparent from the numerous vascular arborizations and the increase of consistence in the effused matter. Well, this rudimentary organization is the first phase of the more advanced ogranization of the same matter, which, whenever it has offered itself during the first period of rachitis under the form of sanguineous effusion, presents itself in the second period under the appearance of a very fine, spongy tissue, more or less abundant and appreciable, and attains a degree of osseous organization more or less advanced; because it is possible, for reasons that I shall mention by and by, for the same subject to offer simultaneously in the different portions of his skeleton, alterations belonging to several periods of the malady. However that may be, the characteristic type of the alterations of texture peculiar to the second period of rachitis, is the presence of a very fine, spongy tissue in every portion of the skeleton, when the affection exists in a sufficiently intense degree. Thus, the short like the long bones, the flat like the purely spongy, present this new tissue in variable degrees. The pelvic bones, the scapulæ, the clavicles, the sternum, the vertebræ, and also the bones of the cranium, contain quantities more or less considerable of this tissue. Any one may assure himself of this by dividing these bones in the fresh and even in the dry state. In the dead body the section of these bones offers ordinarily a greater thickness than in the normal state. It is distinctly seen that this excess of thickness is only due to the presence of a layer of newly formed spongy tissue, more or less thick, which is ordinarily interposed between the periosteum and the bone. This layer is easily distinguished from the old bone by its meshes, which are finer, denser, less resisting and have not so deep a color. Moreover, it can be removed with the greatest facility. In the dry state the recently formed spongy tissue, to which I have given the name of spongoid, in order to distinguish it from the normal tissue, is preserved under the appearance of a contracted layer, of a yellow color, contrasting with the natural color of the bone. This, however, is chiefly discernible in the diaphysis of the long bones.

3 *

I have shown that in the period of incubation the effusion took place between the periosteum and the surface of the bone, between the medullary membrane and the internal osseous layer, and lastly, between the laminæ of the compact tissue, which are divided and exfoliated in some measure; now, the spongy transformation is still more sensible there than at the level of the epiphyses and in the flat and short bones. This newly formed matter is occasionally so abundant that it produces a separation several lines wide between the periosteum and bone, and by means of the unfolding of the lamellæ of the compact tissue, forces the latter back towards the medullary canal, which is considerably lessened, and now and then is entirely obliterated. This peculiarity is remarked particularly when the bones are curved, and is observed on the concave side of the curvatures, whilst on the convex side the laminæ of the compact tissue continue to be closely joined, and the periosteum to be applied immediately upon the bone. This difference between the two sides of the curvature relatively to the presence of the spongoid tissue, seems to be owing to the fact that the effused matter not having been able to make a lodgement between the osseous laminæ aud the periosteum of the convex side, has been forced from the compression of the plates of convexity to flow back between the laminæ and periosteum corresponding to the concavity. There, indeed, this matter has been able to accumulate without obstacle, and to be replaced by the spongoid tissue. Sometimes a lamina three or four lines thick is found between the periosteum and the bone. In this case the former is the seat of a very unusual vascularity, as in the preceding period, and has acquired a consistence and density sensibly exalted. One cannot better assure himself of this than by comparing the periosteum of the two sides of the curvature.

On the convex side it retains nearly the normal conditions; upon the concave side it is evidently hypertrophied, and the spongoid matter deposited at its surface is with difficulty separated. This disposition is present to an unusual degree at the level of the angular curvatures offered by certain long bones, such as the humerus, the femur, &c.

At the same time that the effused matter acquires the characters that I have indicated, the osseous tissue, properly so called, offers some modifications of consistence and structure which it is not less important to describe.

The long bones are bent pretty easily without breaking; sometimes even muscular action suffices to bend them. The flat bones, like those of the cranium and pelvis, suffer themselves to be depressed with the thumb; but for this depression to be possible, the rachitis must have been carried to a very marked degree. A cutting instrument divides them as a somewhat firm cartilage: when fractures accidentally take place in the long bones, they are never complete; a part of the diaphysis has simply bent, at the same time presenting in the longitudinal direction some incomplete fissures. These are the most obvious facts; but they are already sufficient to show that the proper osseous tissue has undergone a certain degree of true softening. By proceeding farther into the examination of the contexture and consistence of the bones, other proofs of this softening are obtained. For instance, the plates that are unfolded and isolated may be reduced to filaments which are twisted and bent in every direction without breaking. Torsion and compression somewhat firm, causes a sanguinolent liquid to escape. When these fragments of lamellæ are placed under the microscope a multitude of small reddish points are perceived dotting, so to speak, the osseous substance itself. These points are particularly appreciable in the most superficial plates of the concave side of the curvature. Lastly, when the compact laminæ, and even the entire bones are dried, then are perceived in the points corresponding to the concavity of the curvatures principally, a multitude of small holes, some of which attest, by their exaggerated development, the proportionate development of the small vessels to which they gave passage.

All these changes that give evidence of an alteration in the consistence of the osseous tissue, properly so called, are not remarked in the long bones solely. In the flat and short bones the external table offers some analogous modifications. As I have already said it gives way to the pressure of the fingers, and is cut with the greatest ease. Sometimes it is rendered considerably thinner by the removal of the internal laminæ. There is no doubt then, that, independently of the formation of a new tissue, the spongoid, characterizing the second period of rachitis, the proper osseous tissue undergoes in this period a true softening.

As it respects the changes of structure, they are not less real, but they are a little different, according to certain conditions which it is important to determine. In the most ordinary cases, the separation of the laminæ by the newly organized rachitic effusion, is the sole operation observed during the second period of rachitis. This separation is a mechanical one, and not the result of an interstitial absorption of the osseous tissue; for the spaces resulting from it are nearly always exactly concentric, and they correspond besides to the separation observed during the first period, when a layer of liquid alone is interposed between these laminæ. There is, therefore, in ordinary cases only a simple unfolding of the concentric layers of the diaphysis, without absorption or interlaminar destruction. At a more decided stage, that is when the effusion has been considerable either at the surface of the bone, between the periosteum and first layer, or between the concentric layers, the texture of the bone proper may offer two kinds of changes. Either the laminæ have been considerably removed from one another and their vascular communications broken and interrupted, in which case the osseous laminæ may detach themselves from the principal layer, fall down in detritus, and constitute a peculiar mode of being of rachitic bones that I have designated and described, as will be seen farther on, under the name of *rachitic consumption;* or else the abundance of spongoid matter deposited at the surface of the concavity of the base forced back the laminæ of the bone proper into the medullary canal, and the laminæ offer a more or less complete solution of continuity at a level with the centre of the effusion, or otherwise only an angular curvature of the bone, whence, in either case, may result an extreme curvature, and also an appearance of incomplete fracture. These modifications are not absolute; on the contrary, they are dependent on some special conditions; and express in no case the general fact of the destruction and disappearance of the proper osseous tissue in the second period of rachitis; the latter result never exists but incompletely and in an accidental manner, and is joined to an extreme degree of the malady, which degree is expressed, as will be seen hereafter, by some consecutive alterations which are completely independent of the most frequent general characters of the rachitic osseous tissue.

### 3.—*Alterations of Texture of the Third Period of Rachitis.*

As the general symptoms of rachitis are dissipated and announce the period of resolution, the osseous tissue follows in its phases an analogous progression and transformation. The spongoid tissue of the epiphyses is in part absorbed, and the remaining portion acquires some consistence and loses some of its flexibility; the liquid which bathed it disappears more and more, its meshes enlarge and solidify. In the body of the bone other changes occur: the interstices resulting from the disunion of the laminæ are filled by true deposits of phosphate of lime. By degrees the lines of separation are effaced, the layer of spongoid tissue which was organized between the periosteum and the bone, and between the medullary membrane and the internal surface of the bone, is transformed successively into compact tissue, and the whole osseous frame-work of the diaphysis offers very soon the homogeneous aspect of the primitive bone. This transformation can be followed with the naked eye in the easiest manner. The interstices of the lamellæ by degrees fill up and contract; the fissures which are at first perceived on the edge of the divided bone very soon offer only some very slender linear appearances betrayed by the reddish color of the spongoid tissue of which some hardly perceptible traces only remain. Lastly, in proportion as the new layers acquire the character and consistence of the compact tissue, this resumes its normal primitive solidity, and even in the end attains an extraordinary hardness and compactness. I have designated this state by the name of *eburnation,* because in fact the rachitic bones offer at this period the aspect and hardness of ivory, and it is impossible, whatever force one employs, to overcome the curvatures without fracturing the bones. This transformation is completed some years after the disappearance of the acute symptoms of rachitis, being the more perfect in proportion to the age of the subject, and vigor of the constitution.

Lastly, in proportion as the period of resolution is accomplished, the large bones which were very crooked, particularly the tibiæ and fibulæ, become flat, and approach the figure of a curved sabre blade. When they are divided longitudinally at this period, nearly an inch of compact bone is sometimes found between the concave border of the bone and the medullary canal. This increase in the breadth of the compact part evidently results from the transformation of the originally spongoid layers of the rachitic bone, confounded with the layers of the old bone. The traces of this effusion can be often recognized by one or more longitudinal lines.

The changes which are brought about during the third period of rachitis, in the flat and short or spongy bones are less remarkable and less absolute. Sometimes the flat bones grow very heavy, hard, and compact, and the spongoid tissue has evidently furnished them the elements of this hyperostosis. The coccygeal bones, for instance, sometimes become of a considerable thickness and hardness. I have observed one pelvis, among others, very curious in this respect, which was shown to me by M. Legrand of La Maternité. It weighed twice as much at least as an ordinary pelvis, and its walls had acquired, in some places, a thickness and compactness triple or quadruple what occur in the natural state. The bones of the cranium also very frequently present the same development: I have seen and described several examples in which the parietal bones particularly had triple the usual thickness. When the cranium of subjects who are in this condition is divided, a multitude of small, reddish, sand-like granulations, very dense and very close, are perceived, which still retain something of the characters of the spongoid tissue. The whole of the diploe is invaded by these granulations, and the entire bone is of remarkable hardness and weight. In the bones entirely spongy, such as the vertebral bodies and the tarsal bones, the characters of the period of resolution also offer some distinct appearances. Their volume does not generally remain augmented, and indeed rarely continues so; but their surface is unequal and rough, and marked with alternate depressions and saliences, which betray an ancient alteration of the consistence of their tissue. Above all the vertebræ are remarkable in this respect; their free surfaces are indented or depressed; one might say that they had been swollen for a moment, and obliged to fall back on themselves by the disappearance or absorption of the matter they contained, that they had sunk and contracted in order to fill the spaces left, and adapt themselves to their actual contents. In all these cases, they are flattened anteriorly or upon the sides to a considerable degree. As to their consistence, it is generally greater: it is frequently almost impossible to divide the bodies of the vertebræ that have been affected with rachitis at a former period. Their compact substance is hypertrophied and formed of a crust of very hard phosphate of lime.

The periosteum in this period is strongly adherent to the bone; it has become again what it was; it is neither thicker nor more injected than in the healthy state, unless upon the concave side where it sometimes preserves a firmer consistence.

Such are the alterations of texture observed during the three periods of rachitis in the young subject, and which constitute the immediate characters of the *recent* affection.

### 6.—*Texture of the Bones in Long-Standing or Chronic Rachitis.*

The texture of the bones in rachitis of long-standing appears under two distinct conditions, according as the disease is completely resolved, leaving no other traces than the deformity of the skeleton, or as the texture of the bones offers some alterations which have persisted after the disappearance of the symptoms of acute rachitis. In the first case, that is, when the disease has ceased for a long time, leaving no other alteration than the distortion of the skeleton, the texture of the bones continues to be what I described it in the third period of rachitis under the name of *eburnation.* The texture of the diaphyses has acquired, in fact, the hardness and density of ivory; all the compact portion of the long bones offers, notwithstanding the very striking curvatures, a homogeniety and compactness which are but the complement of the state that I ascribed to the third period of recent or acute rachitis. The only difference between the old and recent eburnation consists in the rarefaction of certain parts of the osseous tissue. In fact, old rachitic bones perfectly consolidated, present at the level of their epiphyses and in the areolar portion of their diaphyses, large cells which demonstrate the absorption of certain parts, and, furthermore, the diaphysis of the long bones that remain crooked is flattened, as if fallen back upon itself. Save this difference, the ancient eburnation offers the finished characters of the recent, that is to say, the ivory-like portion of the diaphysis, especially that which answers to the concavity of the curvatures, is of an excessive hardness, so much so as to be with difficulty divided with the saw.

When the resolution of the malady has not been complete on account of its intensity, and when, in consequence of a very considerable effusion there has been a separation and detachment of the lamellæ of the compact tissue and excessive dilatation of the areolæ of the spongy tissue, the osseous tissue passes, as I have said, into the state of *rachitic con-*

*sumption.* In this condition, the external layer of the bone, particularly that of the epiphyses and flat bones, is reduced to a very thin, transparent, fragile lamina, yielding to the slightest pressure of the fingers.

The whole space circumscribed by this osseous shell is occupied by large cells which might be said to result from several areolæ united, and in which float more or less freely some remains of the lost lamellæ in the midst of greasy, yellowish marrow, shaded in some places by reddish plates. These cells do not occupy the spongy bones solely, and the extremities of the long bones; they invade the medullary canal of the latter; they are filled, moreover, with fat, and sometimes with particles of lamellæ or with entire isolated lamellæ, which do not appear to have any adhesion with the diaphysis. When these bones are dried, they are reduced to a very thin envelope, semi-transparent and extremely fragile, though bathed almost in an oily matter. The lamellæ which were immersed in the medullary tissue, become free, as if half rolled up, extremely thin and fragile. They appear to have only some imperfect adhesions with the compact tissue. This alteration is not always so striking or so general: frequently the epiphyses alone are affected, and the middle portion of the diaphysis is rendered hard and compact, like ivory; at other times the epiphysis and diaphysis present it simultaneously in a high degree, and a very small effort is sufficient to fracture the bone. According to these two cases, the bones are affected with *complete*, or *incomplete rachitic consumption.*

The mechanism of rachitic consumption seems to me very simple, and consists entirely in a considerable separation of the osseous laminæ to the extent of interrupting their vascular communications, and producing more or less completely the death of the parts. This mode of formation of rachitic consumption can be distinctly verified during the first period of acute or recent rachitis. The bones of young subjects affected with rachitis to this degree present a remarkable exfoliation of their tissue, so that the cortical layer of all the bones is reduced to a simple pellicle. I have several skeletons of young subjects in which this exfoliation of the compact tissue is evident. One may conceive that the parts thus violently disjoined cannot, when the malady subsides, reunite, and that the rachitic effusion, unprovided with vascular communications, cannot organize itself and assume successively the characters of the osseous tissue, without the elements which should come from the periosteum or the medullay membrane.

Such are my observations touching the different alterations of texture proper to the different phases of rachitis; they may be thus recapitulated:

1. The texture of rachitic bones offers characters altogether different according as they are observed during the period of *incubation, of deformity, or of resolution*, varying at the *commencement and the end* of each of these periods, and, lastly, according to the degree of antiquity of the affection.

2. During the period of incubation an effusion of sanguinolent matter occurs in all the interstices of the osseous tissue, in the cells of the spongy tissue, the medullary canal, between the periosteum and the bone, between the concentric lamellæ of the diaphysis, between the epiphyses and diaphyses, between the epiphysary nuclei and their cellules, in the short and the flat as well as in the long bones, in a word, in every portion of the skeleton, and every point of the osseous tissue where the radicles of the nutritive vessels are distributed. From this effusion come the exfoliation of the component parts of the tissue, and the swelling and enlargement of the different portions of the skeleton.

3. During the second period of rachitis, that of deformity, at the same time that the osseous frame-work loses its consistence and grows soft, the matter which continues to be deposited in all the interstices of the bony tissue, has at endency to organize; it passes successively from the cellulo-vascular to the cellulo-spongy state. This newly formed matter is particularly abundant between the periosteum and the bone, the medullary membrane and the canal, the periosteum and external table of the flat bones, and between the plates of the latter.

4. During the third period, that of resolution, the recently formed tissue in the long bones, and in some of the flat and short ones, passes to the state of compact tissue, and tends to confound itself with the old tissue which recovers its former hardness. This addition of a new tissue to the old one gives a very great thickness, and especially a very great breadth, to some parts of the bones which had been the seat of the organization of the spongy tissue of the preceding period.

5. In the state which I have designated under the name of rachitic consumption, and which results from an exaggerated degree of the affection, the unfolding and the separation of the component parts of the osseous tissue have been such that their re-union is not effected, and the organization of the effused matter does not take place. In this state the partitions and the osseous lamellæ are left separated, and the consistence of the primitive bone has been reduced so much that the external layer is sometimes formed by a thin pellicle only.

6. The texture of rachitic bones among adults, when the affection is completely resolved, offers a compactness and a hardness superior to those of the normal state. In this state which I call that of rachitic eburnation, no trace of the union of the elements of the old with those of the new bone can any longer be distinguished.

*c.—Influence of Rachitis upon the Progress of Ossification.*

The authors who have occupied themselves with the question, whether rachitis exercised any influence whatever upon the progress of ossification, have frequently decided it in a contradictory manner: some have said that the work of ossification had seemed to them to continue as under ordinary circumstances, others that the softening itself of the osseous tissue was a condition that necessarily retarded its consolidation; this diversity of opinion is owing to a want of precise observation, and arises from investigations made under different circumstances.

The appreciation of the influence of rachitis upon the progress of ossification can arise, then, in a complete manner only from an examination of the osseous tissue in the different periods of the malady, and at greater or less distances from its invasion; moreover, the facts which express this influence are not all of the same order; they belong to distinct categories: some relate to the state of ossification, properly so called, that is, to the transformation of the cartilaginous tissue into the osseous; others to the mode of junction of the epiphyses, and to the degree of soldering of the parts originally separate and distinct.

Respecting the work of ossification, properly so called, it seems difficult at first view, confining oneself solely to the external phenomena, to distinguish what is the effect of the delay of ossification from what belongs to morbid softening. For instance, the sternum and ribs are seen to preserve such a suppleness and elasticity during the existence of rachitis that they can be indented with the fingers with the greatest ease. It happens even that the sternum may bend double and form a curvature re-entering from above downwards. Are these results owing to a morbid softening, or rather to the effect of a delay in the ossification? I think that they are ascribable at once to both of these causes. To be convinced of this it is sufficient to compare the state of the osseous tissue in rachitic subjects with that of healthy persons of the same age. This inspection has shown me that in rachitic individuals during the second period and at the commencement of the third, a greater number of filaments or cartilaginous nuclei are distinguished in the midst of the proper osseous tissue. The cartilaginous filaments are perceived more easily in the vertebral bodies, the cartilaginous nuclei in the epiphyses. The latter present themselves under the form of small rounded bodies separated from the principal nucleus, and lost as it were in the spongy substance; their color is less distinct being slightly bluish; they are, moreover, rather slightly united to the spongy substance; when detached with the point of the scalpel they have in the place they occupied an excavation analogous to that left by tubercles of the bones. In regard to the mode and degree of union of the epiphyses with the diaphysis of the long bones, there is from all the evidence a marked delay in rachitic individuals. The divulsion of the epiphyses is effected with the greatest facility, and effused blood is found, during the first period of rachitis, at the point of union between the epiphysis and diaphysis, like what is found between the periosteum and the compact tissue of the diaphysis. This phenomenon becomes very sensible when a rachitic skeleton is dried; all the epiphyses of the bones of the extremities come off from the diaphysis with the utmost ease. This disposition accounts well for the coming off of the epiphyses, which several authors say they have observed following certain eruptive diseases: now it is known that in these circumstances the blood has often lost its plasticity, and that it is under the influence of the same circumstances, as I shall demonstrate by and by, that rachitis is developed.

Lastly, some analogous effects are remarked in the bones originally composed of distinct pieces, such as the sternum, the os coccygis, &c.; the pieces of these bones appear to unite more slowly than in the healthy state. Those of the sternum, for example, show this effect in a very high degree. Mostly the different portions of which it is composed remain divided, and the separation is perpetuated during life. One can assure himself of this fact on nearly all the skeletons of adults who offer marks of a very high degree of rachitis. The result of this separation is an anormal projection of the sternum at the point of union of the portions composing it. The same effect may again be observed on the os coccygis, but in a less striking manner, because the ossification of its several portions is accomplished much later. Yet in their degree of disjunction and the facility with which they are separated, it is impossible not to recognize one of the results of the influence of rachitis.

The final or collective result furnished by these two orders of facts, namely, the persistance of a more marked number of cartilaginous nuclei, the separation of the epiphyses, and the more tardy union of the portions composing the multiple bones, show, without any doubt, a delay in the work of ossification.

Such is the series of facts constituting the purely anatomical history of rachitis. The greatest part of them are new, and the result of my own researches: some of their elements have been indicated, but indicated only, and in a more or less incomplete manner, by those who have preceded me. Among these I will cite particularly Bichat, Lévillé, Stanley, Geursant, Rufz, and Dugés, the only ones who have published any observations deserving attention on the pathological anatomy of rachitis. Bichat, for instance, has noticed in the middle portion of the long bones in rachitic individuals an osseous areolar-like tissue, which he considers, however, as resulting from the extension and separation of the osseous fibres.* Léveillé is of the same opinion. We are indebted to Rufz for some observations much more precise and detailed.† This author has remarked the lamellar disposition of the long bones and the presence of a reddish medullary substance deposited between each of these lamellæ, as well as between the cells of the spongy substance, and the enlargement of the epiphyses by the presence of a newly formed spongy tissue. But besides that he does not appear to have conjectured the connection of these alterations and the other transformations anterior and posterior, corresponding to the different periods of the malady, he has circumscribed in these two facts, considered absolutely, all the alterations of texture of rachitic bones, forgetting that such of the alterations as he had noticed in a single point of the long bones, namely, the existence of a new spongy tissue at the union of the epiphyses with the diaphysis, is a general fact, that is found in every part of the skeleton, at the surface and in the interior of the long bones throughout their entire length, at the surface of the flat bones as well as between the laminæ of

* Bichat, Anatomie Génerale, edit. by Chaudé, t. iii. p. 31.

† Gazette Médicale, 1834, pp. 68, 69.

their table; in every place, in a word, where I have desbribed it, as a consequence of an alteration of ossification, as succeeding to the general effusion of the first period of rachitis, and as becoming the ground-work of supplementary ossification of the long, and sometimes of the flat bones.

An English author, Stanley, has published some remarks upon the state which the long bones, affected with rachitis, present towards the termination of this affection, and whilst showing the usual development of the compact tissue at the concavity of the curvations, he attributes this development, like M. Rufz,* to hypertrophy of the normal tissue, without referring this transformation of the osseous tissue to the anormal phases preceding it.† Lastly, M. Dugés, has adopted and confirmed, in part, the researches of Rufz and Stanley. But any one may see, that these authors have all perceived but one or more isolated points of the pathological anatomy of rachitis, without having a glimpse of the origin, the connection, and the order of succesion that these phenomena have with each other; without even recognizing the actual universality of the facts of which they have remarked some local manifestations. Now the character of true observation is not solely to verify some peculiarities of phenomena, to perceive them in one point of their extent, but to seize them in their totality, to show their origin, their mode of evolution, of succession and of association, to follow them in their succession

---

* Loc. cit.

† The English author, moreover, considers the enlargement of rachitic bones at the level of their ancient curvations, as resulting from the provision of nature, which endeavors to fortify the parts having the most efforts to bear. "Thus," says Stanley, "it is evident that in the crooked bone, the place where there is most need for strength to prevent it from giving way more is the middle of its concavity, and it is in this situation that the bone will receive some strength and compactness by the deposition of phosphate of lime." (*Medico-Chir. Trans.* vol. viii. p. 104.)

and their relations, to point out, in a word, their signification and respective importance; were it not for this, the least practised eye might perceive one or several isolated points of the most beautiful discoveries, one or more links of the chain which runs back to the point of departure, that is to say, to the cause: but he would discern only these separate links, and would not lay hold of their connections with those that precede or follow them, and, above all, would not attain to the general signification of the facts of which he could embrace only a few material circumstances.

### Sec. IV.—*Diagnosis of Rachitis.*

After having made known in detail the characters of genuine rachitis, considered in its general phenomena and in each of its particular elements, I have still, in order to complete the description of this malady, to compare it with the morbid forms to which the same denomination has improperly been given. The contrast that must result from this comparison will have the effect, I trust, of destroying the surperficial analogies which have been traditionally admitted.

The diseases with which rachitis has for a long time been confounded are most of the *deformities of the spine, the tuberculous affection of the bones, and the osteomalacia of different species.*

The spinal deformities improperly ascribed to rachitis are all lateral deviations, of whatever nature they may be, and the excurvations, particularly those of the tuberculous kind. This confusion would appear no longer possible, particularly with respect to tuberculous affections of the vertebral column; nevertheless, it still exists even in the most recent works. Thus, in a treatise on the diseases of the spinal marrow, the terms *caries of the vertebral column*, *Pott's disease*, and *rachitis*, are indifferently employed to designate the same malady. It will not be superfluous, therefore, to describe these vexatious synonymes.

The lateral deviations of the spine will no longer be attributed exclusively to rachitis, because the greatest part of these deformities are the result of other well established causes, and because true rachitis, pretty rare withal, especially in the wealthier classes, is accompanied by circumstances and characters which make it easily understood. It is sufficient to call to mind that every rachitic deformity of the spine has necessarily been preceded by some general symptoms of rachitis, and particularly by deformities of the inferior members. Consequently every deformity of the spine wanting this accompaniment, in an immense majority of cases at least, is not of a rachitic nature, and cannot be confounded with rachitis. In a particular memoir upon rachitic deviations of the spine, I shall specify the characters peculiar to that species of deviation. Let us add that the osseous tissue of the spinal columns affected with lateral deviations without the external characters which I have just recalled, never presents the modifications of texture so characteristic of rachitis. The vertebræ offer no other alterations than those resulting from their changes of relation, and from the irregular mechanical efforts to which they are subjected, comprehending in these the influence of the degree and of the duration of action of these influences, and that more general one of the deformity of the spine upon the organism, and the effect of this reaction upon the ensemble of the osseous tissue.

Tuberculous excavations and the tuberculous affection of the bones will no longer be referred to, and confounded with rachitis. The tuberculous affection of the bones and rachitis are, in fact, two essentially distinct diseases which are encountered sometimes in the same subject, but have very different causes, symptoms, and anatomical characters, as well as very different general physiognomy. Tuberculous excurvations are never preceded or accompanied by deformities of the inferior members, unless true rachitis itself has existed, which I have sometimes seen. But the simultaneous occurrence of two different affections so easy to distinguish does not imply an identity in their nature; and their peculiar characters, though existing simultaneously in the same individual, can be recognised as if they existed separately. There is no necessity of my setting forth the contrasts resulting from the age of invasion, from the progress, or from the treatment of these two diseases. I shall limit myself to recalling the most prominent characters of the osseous tissue in the tuberculous affections which bring in their train certain deformities of the spine. Now, the bones seized with the tuberculous affection are nearly always the spongy bones, or the extremity of the long bones. The affection is never announced with a character of generality like rachitis; it shows itself, on the contrary, in certain circumscribed points of the skeleton, and the parts affected by it, as the vertebræ and epiphyses of the long bones, never offer the succession of phases so distinct of the rachitic alterations; finally, the tubercles, which have been justly regarded as parasitic bodies, invade successively the different portions of the bone where they are developed, destroying them rather mechanically than chemically, and leaving often to the preserved portions of this tissue, but adjoining the altered points, all their hardness, and all the dispositions of their primitive texture. Any one may easily convince himself of this fact by washing in a large quantity of water some vertebræ riddled with tubercles: the remaining portions offer very nearly the characters of the most healthy tissue. It might appear no longer necessary to mention these peculiarities for those who follow closely the progress of the science; but besides that they are too little known by the greater number, I have some reasons for establishing, with all possible precision, that rachitis is anatomically wholly different from the tuberculous affection of the bones: for, in my experiments upon the production of spontaneous rachitis in animals, it has happened to me to have apparently very little change to make in order to produce the one or the other of these two affections.

With more appearance of reason the various species of softening of the osseous tissue in adults have been confounded with rachitis exclusively proper to children; since the history of Mad. Supiot the denomination of rachitis has even been retained for them. That which more than any thing else has tended to perpetuate this error, is that the best authors who have described rachitis, as Glisson, have not entirely escaped the confusion of terms. It is proved to my satisfaction, however, that these two orders of diseases, the softening of the osseous tissue in adults, and rachitis as I have described it, can no longer be attributed to the same cause, that they offer neither the same characters, nor the same symptoms.

I have already shown that true rachitis is a disease of infancy, being observed chiefly towards the age of eighteen months or two years; that it is characterized by a mode of invasion, a course, periods and alterations of texture altogether peculiar; now in the different species of softening, observed in adults, like that of which the woman Supiot offers a very beautiful example, there is no analogy between these various terms of comparison. Although it is not my intention to insist here upon the detailed history of softening of the bones among adults, which I propose to do shortly, it will be sufficient for me to call to mind the principal circumstances of the malady in order to prevent all ulterior confusion.

The softening of the bones in adults to which I restrict the term *osteomalacia*, is the result of specific causes, such as scurvy, syphilis, rheumatism, or of some particular vice, as the cancerous: every individual who has offered it, had presented, prior to the commencement of the softening, the general symptoms of these alterations. It is announced by lively and deep-seated pains in the bones; the progress of the malady is slow; it endures for a great many years, sometimes for 20 years; it does not declare itself simultaneously in every portion of the skeleton, nor from below upwards, but it attacks it by fractions only, so that when an individual is examined who presents it in a degree as yet but little advanced, some bones are found affected singly, and even portions of bone altogether softened at the side of other portions of the same bone retaining their normal resistance and texture. I have in my possession several examples of this partial softening observed in persons dying with cancers of the stomach, breast, or uterus. I shall make them known hereafter in detail. If the nature of the alteration of the tissue be examined closely, this inspection alone will convince any one that osteomalacia and rachitis are two essentially different affections. In the one, the osseous tissue is truly softened, as if carnified in places, and no longer retains any thing of the consistence or of the texture of the healthy bone; it is as if one had poured upon the seat of the softening a very powerful liquid, having the property of causing immediately every trace of calcareous salts to disappear, so as to leave nothing but a fibro-cartilaginous or even fleshy net-work, presenting here and there layers of areolæ like the venous sinuses of the liver; this net-work is sometimes yellow or rosy, sometimes reddish, or of the color of wine-lees, always elastic, very easily cut with the knife, but sometimes incrusted as it were with some other portions of the sound tissue. This circumscribed state of the disease is far from being constant; at a more advanced period, it often happens that the skeleton partakes of the softening, and, as is seen from reported observations of authors, there no longer remains any appearance of the primitive organization of the bones. The termination of the osteomalacia, which is always unfortunate, adds another to the distinguishing traits between it and rachitis.

It will be seen, therefore, that the deformities of the vertebral column, considered in an essential manner, tuberculous excurvations of that portion, or the tuberculous affection of the bones, and osteomalacia, constitute affections entirely different from rachitis, and which will not henceforth be confouneded with it.

### Sec. V.—*General Summary and Conclusions.*

The researches contained in this memoir may be recapitulated as follows:

1. Rachitis is a general affection of infancy, characterized by the alteration or perversion, and also the suspension of the process of development and of reparation of the organism, and particularly of the osseous system.

2. The progress of rachitis, considered as an affection of the skeleton, comprises three distinct periods: first, the period of *incubation* or of *effusion;* second, that of *deformity;* and third, that of *resolution* or *eburnation*; to each of these periods correspond some peculiar general symptoms, and some peculiar alterations of the osseous tissue.

3. The influence of rachitis upon the osseous tissue is revealed by four distinct orders of facts: first, the *deformity;* second, the *alteration of tissue;* third, the *arrest of development;* and fourth, the *delay of ossification.*

4. The rachitic deformity of the skeleton is developed successively from below upwards, from the bones of the leg to the thigh bones, from these to the pelvis; then come successively or simultaneously the different portions of the superior limbs, the thorax, and, in the last place, the vertebral column and the cranium.

The degree of the deformities is in relation with their order of development; whence it follows that the rachitic deformity of one portion of the skeleton always implies that of the portions situated below.

5. Most of the bones of the rachitic skeleton are always relatively less developed in length and breadth than those of the healthy skeleton; this shortening which is independent of that resulting from the deformities is effected according to the same laws; that is to say, successively from below upwards, and gradually from above downwards. The proportion according to which all these parts of the skeleton are reduced from below upwards is expressed by a regular series of numbers, which permits us from the dimensions of a single bone to deduce approximately that of the other portions of the skeleton.

6. The greatest reduction of the inferior compared to that of the superior members establishes between these parts relations of length, which repeat and perpetuate those of the age at which the malady is developed.

7. The shortening of the bones considered in rachitic

TABLE No. I.

### COMPARATIVE TABLE OF THE DIFFERENT PARTS OF THE RACHITIC AND HEALTHY FEMALE SKELETON.

| DESIGNATION OF PIECES. | | FIBULA. | TIBIA. | FEMUR. | RADIUS. | ULNA. | HUMERUS. | CLAVICLE. | STERNUM. | VERTEBRAL COLUMN. | DIAMETERS OF THEP ELVIS. | | | | PERIMETERS OF THE CRANIUM. | | |
|---|---|---|---|---|---|---|---|---|---|---|---|---|---|---|---|---|---|
| | | | | | | | | | | | ANTERO-POSTERIOR. | OBLIQUE. | TRANSVERSE. | TOTAL. | HORIZONTAL. | From one auricular canal to the other, passing over the vertex. | From the nasal spine to the occipital protuberance. |
| | IN. L. | IN. L. | IN. L. | IN. L. | IN. L. | IN. L. | IN. L. | IN. L. | IN. L. | IN. L. | IN. L. | IN. L. | IN. L. | IN. L. | IN. L. | IN. L. | IN. L. |
| Dupuytren Museum. 338 . . . | 43. 6 | 9. 9 | 11. 7 | 13. “ | 7. 1 | 8. 1 | 10. “ | 5. 5 | 5. “ | 17. “ | 4. “ | 4. 9 | 4. 7 | 13. 4 | 19. 8 | 12. 4 | 11. 10 |
| Maternité. 335 . . . . . . . . . . | 43. 3 | 7. 1 | 6. 6 | 8. 5 | 4. “ | 5. 3 | 5. 9 | 4. 5 | 4. 5 | 20. “ | 1. 11 | 3. 11 | 4. 3 | 10. 1 | 20. 9 | 14. 3 | 13. 3 |
| Muette. 24 . . . . . . . . . . . . . | 48. “ | 11. 3 | 12. 1 | 14. 4 | 7. 6 | 8. 3 | 10. 9 | 5. 8 | 4. 9 | 20. 10 | 2. 7 | 4. 5 | 5. 3 | 12. 3 | 20. “ | 11. 6 | 11. 9 |
| Clamart. 4 . . . . . . . . . . . . . | 45. 6 | 9. 6 | 10. 8 | 10. 11 | 6. 4 | 7. 2 | 9. 5 | 5. 2 | 4. 9 | 26. 5 | 2. 7 | 4. 3 | 5. 1 | 11. 11 | 19. “ | 12. “ | 11. 3 |
| do. 26 . . . . . . . . . . . . | “ “ | 9. 9 | 10. 7 | 10. 8 | 7. 7 | 8. 3 | 9. 10 | “ “ | 5. 9 | 21. 6 | 2. 6 | 4. 9 | 5. 6 | 12. 9 | “ “ | “ “ | “ “ |
| do. 16 . . . . . . . . . . . . | “ “ | 11. 11 | 13. 2 | 14. 2 | 8. 8 | 9. 9 | 12. “ | “ “ | 5. 3 | 22. 6 | 2. 4 | 4. 6 | 4. 9 | 11. 7 | “ “ | “ “ | “ “ |
| do. . . . . . . . . . . . . . . . . | “ “ | 10. “ | 11. 2 | 10. 11 | 7. 6 | 8. 3 | 8. 7 | “ “ | “ “ | 20. “ | 3. 7 | 4. 6 | 5. “ | 13. 1 | “ “ | “ “ | “ “ |
| do. 30 . . . . . . . . . . . . | 32. “ | 8. 1 | 9. 3 | 9. 11 | 5. 10 | 6. 9 | 7. 10 | “ “ | “ “ | “ “ | 2. “ | 3. 3 | 3. 3 | 8. 6 | 17. “ | 12.10 | 13. 2 |
| Dupuytren Museum. F. . . . . | “ “ | 11. 5 | 12. 2 | 11. 6 | 7. 5 | 8. 5 | 9. 7 | 5. 2 | 6. “ | 23. 3 | 3. 9 | 5. “ | 5. 3 | 14. “ | 19. 3 | 12. 4 | 11. 3 |
| do. . . . . . . . . . . . . . | 37. 6 | 7. 11 | 10. 9 | 10. 11 | 5. 4 | 5. 9 | 7. 4 | 4. 7 | 4. “ | 21. 3 | 2. 9 | 3. 10 | 4. 9 | 11. 4 | 18.10 | 12. 3 | 12. 2 |
| do. D. 4. . . . . . . . . | 35. 6 | 8. 9 | 9. “ | 9. 9 | 5. 9 | 6. 5 | 8. “ | 5. 2 | 4. 6 | 20. 11 | 2. 7 | 3. 11 | 4. 3 | 10. 9 | 18. 1 | 12. 3 | 10. 9 |
| do. deposited by Dalmas . . . | 35. 3 | 6. 1 | 6. 11 | 7. 5 | 6. 2 | 6. 7 | 6. 6 | 4. 11 | 5. 1 | 20. 9 | 2. 10 | 3. 6 | 3. 9 | 10. 1 | 19. “ | 13. 3 | 12. 8 |
| do. . . . - . . . . . . . | 44. “ | 10. 7 | 11. 2 | 10. 1 | 7. 10 | 8. 2 | 10. “ | 5. 11 | 5. 8 | 22. 6 | 2. 11 | 5. “ | 5. 3 | 13. 2 | 19. 9 | 12. 4 | 12. 3 |
| Rachitic Mean . . . . . . . . . . | 40. 6 | 9. 5 | 10. 1 | 10. 11 | 6. 8 | 7. 6 | 8. 11 | 5. 2 | 5. “ | 20. 11 | 2. 10 | 4. 3 | 4. 8 | 11. 9 | 19. 1 ½ | 12. 6 ½ | 12. “ |
| Normal Dimensions . . . . . . | 60. “ | 13. 2 | 13. 6 | 14. “ | 8. 4 | 9. 3 | 10. 6 | 5. 8 | 5. 5 | 22. “ | 4. “ | 4. 6 | 5. “ | 13. 6 | 19. “ | 12. 3 | 11. 6 |
| Ratio of the Rachitic Mean with the Normal Dimensions . . . . . . . . . . . . . . . . . . | 0.67 ½ | 0. 72 | 0. 75 | 0. 78 | 0. 80 | 0. 81 | 0. 85 | 0. 91 | 0. 92 | 0. 95 | | 0. 87 | | | 1.01 | 1.02 | 1. 04 |

### RATIO BETWEEN THE LENGTH OF THE SUPERIOR AND INFERIOR LIMBS IN THE RACHITIC AND IN THE WELL-FORMED FEMALE.

| | RADIUS AND HUMERUS. | FEMUR AND TIBIA. | RATIO. |
|---|---|---|---|
| Healthy adult female . . . . . . . | 18. 10 | 27. 6 | 1 : 1. 47 |
| Rachitic do. do. . . . . . . . . | 15. 7 | 21. “ | 1 : 1. 35 |

adults is a result composed of the arrest of development of the osseous system under the direct influence of the disease, and of the consecutive slowness of its increase subsequent to the disease.

8. The texture of rachitic bones presents entirely different characters according as they are observed during the period of *incubation*, *deformity*, or that of *resolution ;* they differ at the beginning and the end of each of these periods, and are different, lastly, according to the *degrees* and the *age* of the affection.

9. During the period of incubation an effusion of sanguinolent matter occurs in all the interstices of the osseous tissue, the cells of the spongy tissue, the medullary canal, between the periosteum and the bone, the concentric lamellæ of the diaphyses, the epiphyses and the diaphyses, the epiphysary nuclei and their cells, in the short and the flat, as well as in the long bones; in a word, in every part of the skeleton, and all the points of the osseous tissue to which the radicles of the nutrient vessels are distributed. From this effusion result the exfoliation of the parts composing the tissue, and the swelling and enlargement of the different portions of the skeleton.

10. During the second period, that of deformity, at the same time that the frame-work of the osseous tissue is losing its consistence and growing soft, the matter which continues to be deposited in all the interstices of this tissue tends to organization: it passes successively from the cellulo-vascular to the cellulo-spongy form. This newly formed matter is particularly abundant between the periosteum and bone, the medullary membrane and the canal, the periosteum and external table of the flat bones, and between the plates of the latter.

11. During the third period, that of resolution, the newly formed tissue in the long, and in some of the short and flat bones, passes into the condition of the compact tissue, and has a tendency to confound itself with the old tissue, which retains its primary hardness. This addition of a new to the ancient tissue gives a very great increase of thickness, and especially of width to some parts of the bones which had been the seat of the organization of the new spongy tissue of the preceding period.

12. In the state I have designated under the name of *rachitic consumption*, and which results from an exaggerated degree of the affection, the unfolding and the separation of the parts composing the osseous tissue, are so great that their reunion has not been effected, nor the organization of the effused matter taken place. In this condititïon the partitions and osseous lamellæ have continued separate, and the consistence of the primitive bone has been so much reduced that its external layer is formed sometimes by nothing more than a thin pellicle.

13. The texture of the rachitic bones in adults when the disease has been completely dissipated presents a compactness and hardness superior to that of the healthy state. In this state, which I call *rachitic eburnation*, one can no longer discern any trace of the union of the elements of the old with those of the new bone.

14. The deformities of the spine, which happen towards the age of puberty, and all those which have not been preceded by deformities of the inferior limbs are not of a rachitic nature.

15. Rachitis is an affection essentially different from *scrofula*, or from the *tuberculous* affection of the bones, as well as from every species of *softening of the bones observed in adults ;* for these latter affections the denomination of *osteomalacia* must be exclusively reserved.

### TABLE No. II.

### DIMENSIONS OF THE DIFFERENT PARTS OF THE SKELETON IN RACHITIC CHILDREN.

| SEX AND AGE. | WAIST. | PERONEUS. | TIBIA. | FEMUR. | RADIUS. | CUBITUS. | HUMERUS. | PERIMETERS OF THE CRANIUM | | |
|---|---|---|---|---|---|---|---|---|---|---|
| | | | | | | | | HORIZONTAL. | From one auricular canal to the other, passing over the vertex. | From the nasal spine to the occipital protuberance |
| | IN. L. | IN. L. | IN. L. | IN. L. | IN. L. | IN. L. | IN. L. | IN. L. | IN. L. | IN. L. |
| Girl.... aged 2 years.... | 18. 4 | 4. 6 | 4. 9 | 5.11 | 3. 6 | 3. 10 | 4. 2 | 18. 6 | 12. 3 | 12. 3 |
| do.... " 2½ " ... | 26. 6 | 4.11 | 6. 3 | 5. 6 | 4. 1 | 4. 4 | 4. 3 | 19. " | 12. " | 12. " |
| do.... " 2 " ... | 19. 10 | 4. 5 | 4. 10 | 5.11 | 3. 4 | 3. 9 | 4. 2 | 17. 9 | 13. 2 | 13. " |
| do.... " 3 " ... | 17. 6 | 4. 8 | 5. 1 | 4. 9 | 3. 7 | 4. 1 | 3. 5 | 22. 2 | 15. 2 | 14.11 |
| Boy.... " 3 " ... | 29. 6 | 5. 6 | 5. 9 | 6.10 | 4. 1 | 4. 9 | 4. 8 | 18. 3 | 12. 2 | 12. 5 |
| Rachitic Mean........... | 22. 4 | 4. 9½ | 5. 4 | 5. 9½ | 3. 8½ | 4. 2 | 4. 1½ | 19. 1½ | 12.11½ | 12.11½ |
| Normal Dimensions....... | 29. 1 | 5. 4½ | 5. 9 | 6. 6 | 3.11 | 4. 4 | 5. 3 | 17. 6 | 11. 5½ | 11.11 |
| Ratio of the Rachitic Mean with the Normal Dimensions............... | 0. 76 | 0.89 | 0. 91 | 0.89 | 0.95 | 0. 96 | 0.79 | 1.09 | 1.13 | 1.09 |

### TABLE No. III.

### DIMENSIONS OF THE NORMAL SKELETON IN CHILDREN FROM ONE TO THREE YEARS OLD.

| SEX AND AGE. | WAIST. | FIBULA. | TIBIA. | FEMUR. | RADIUS. | CUBITUS. | HUMERUS. | PERIMETERS OF THE CRANIUM | | |
|---|---|---|---|---|---|---|---|---|---|---|
| | | | | | | | | HORIZONTAL. | From one auricular canal to the other, passing over the vertex. | From the nasal spine to the occipital protuberance. |
| | IN. L. | IN. L. | IN. L. | IN. L. | IN. L. | IN. L. | IN. L. | IN. L. | IN. L. | IN. L. |
| Boy.... 20 months old... | 25. " | 4. " | 4. 7 | 5. " | 3. 2 | 3. 4 | 4. " | 16. " | 11. 3 | 12. 3 |
| do.... 2 years "... | 28. 4 | 5. " | 5. 3 | 6. " | 3. 6 | 4. " | 4. 5 | 16. 9 | 11. 9 | 12. 6 |
| Girl.... 2½ " "... | 29. 1 | 5. 4 | 5. 7 | 7. " | 3. 9 | 4. 2 | 5. " | 18. " | 11. 6 | 11. " |
| do.... 2½ " "... | 35. 1 | 6. 5 | 6. 9 | 7. 8 | 4. 7 | 5. 1 | 6. 3 | 18. 6 | 12. 6 | 12. " |
| do.... 2 " "... | 29. " | 6. 3 | 6. 6 | 6. 11 | 4. 1 | 4. 9 | 5. 10 | 18. 2 | 11. 6 | 13. " |
| do.... 20 months old... | 28. " | 5. 3 | 5. 9 | 6. 3 | 4. 3 | 4. 10 | 5. 10 | 17. 6 | 10. 3 | 10. 9 |
| Mean Length........... | 29. 1 | 5. 4½ | 5. 9 | 6. 6 | 3. 11 | 4. 4 | 5. 3 | 17. 6 | 11. 5½ | 11. 11 |

### RELATIONS OF THE SUPERIOR TO THE INFERIOR LIMBS:

Radius and Humerus.... 9*in.* 2*l.* Femur and Tibia.... 12*in.* 3*l.*—Ratio.... 1 : 1. 34

